# Supplementary material for: Unveiling the “Veil” of information disclosure: Sustainability reporting “greenwashing” and “shared value”
Source: PLoS One. 2023 Jan 18;18(1):e0279904. doi: 10.1371/journal.pone.0279904 (PMC9847897; doi:10.1371/journal.pone.0279904)
Supplement: S1 Table — (DOCX) [file pone.0279904.s001.docx]

**S1Table. Sustainability report “greenwashing” indicator system supplemental information.**

| **Category** | **Indicator** | **Quantized contents** | **Symbolic disclosure** | **Substantive disclosure** |
| --- | --- | --- | --- | --- |
| Readability | Report form | Separate disclosure of sustainability reports | Non-disclosure | Disclosure |
|  | standardization | The report has a cover, table of contents, illustrations and descriptions | Non-disclosure | Disclosure |
| Reliability | Third-party audits or evaluations | Independent third-party audit or evaluation | Non-disclosure | Disclosure |
|  | Whether to refer to GRI | Refer to the GRI Sustainability Guidelines | Non-disclosure | Disclosure |
| Completeness | Shareholder interests | Information transparency, dividends, value preservation and appreciation, and protection of the rights and interests of small and medium-sized shareholders | Text qualitative description | Number quantitative description |
|  | Creditor interests | Debt solvency, information transparency | Text qualitative description | Number quantitative description |
|  | Employee interests | Good remuneration and benefits, learning and development opportunities, physical and mental health development | Text qualitative description | Number quantitative description |
|  | Supplier interests | Establish a level playing field and share the fruits of development | Text qualitative description | Number quantitative description |
|  | Customer interests | Provide quality service to meet customer requirements | Text qualitative description | Number quantitative description |
|  | Environmental responsibility | Reduce pollutant emissions, increase investment in environmental protection, and develop clean energy and renewable energy | Text qualitative description | Number quantitative description |
|  | Financial responsibility | Operating performance and financial performance, capital operation, resource integration | Text qualitative description | Number quantitative description |
|  | System building and corporate governance | Enterprise organizational structure, management system | Text qualitative description | Number quantitative description |
|  | Safety | Safety performance indicators, safety hazard management | Text qualitative description | Number quantitative description |
|  | Public activities | Public Relations and Public Welfare | Text qualitative description | Number quantitative description |
|  | Sustainable development | Enterprise core value culture, development strategy, mission and vision | Text qualitative description | Number quantitative description |
|  | International exchange | Carry out international cooperation and exchange of energy and environmental technology | Text qualitative description | Number quantitative description |
|  | Description of the defect | Sensitive topics and problems and flaws of the enterprise | Text qualitative description | Number quantitative description |
