# Supplementary material for: Unveiling the “Veil” of information disclosure: Sustainability reporting “greenwashing” and “shared value”
Source: PLoS One. 2023 Jan 18;18(1):e0279904. doi: 10.1371/journal.pone.0279904 (PMC9847897; doi:10.1371/journal.pone.0279904)
Supplement: S2 Table — (DOCX) [file pone.0279904.s002.docx]

**S2Table. Correlation coefficients.**

|  | **CSV** | **GI** | **SIZE** | **ROA** | **LEV** | **GROWTH** | **TAT** | **TOP1** | **BZ** | **IDR** | **SZ** | **AGE** | **Market** | **GDP** |
| --- | --- | --- | --- | --- | --- | --- | --- | --- | --- | --- | --- | --- | --- | --- |
| CSV | 1 |  |  |  |  |  |  |  |  |  |  |  |  |  |
| GI | -0.152*** | 1 |  |  |  |  |  |  |  |  |  |  |  |  |
| SIZE | 0.297*** | -0.379*** | 1 |  |  |  |  |  |  |  |  |  |  |  |
| ROA | -0.107*** | -0.0130 | -0.058*** | 1 |  |  |  |  |  |  |  |  |  |  |
| LEV | 0.244*** | -0.132*** | 0.511*** | -0.374*** | 1 |  |  |  |  |  |  |  |  |  |
| GROWTH | -0.0100 | 0.022** | -0.00400 | 0.107*** | -0.053*** | 1 |  |  |  |  |  |  |  |  |
| TAT | 0.037*** | -0.077*** | 0.00600 | 0.086*** | 0.072*** | 0.00300 | 1 |  |  |  |  |  |  |  |
| TOP1 | 0.117*** | -0.076*** | 0.288*** | 0.062*** | 0.092*** | 0.0100 | 0.068*** | 1 |  |  |  |  |  |  |
| BZ | 0.163*** | -0.113*** | 0.334*** | -0.038*** | 0.215*** | -0.0120 | 0.0120 | 0.064*** | 1 |  |  |  |  |  |
| IDR | -0.039*** | 0 | 0.00400 | -0.0120 | 0.00100 | 0.00200 | -0.00500 | 0.048*** | -0.442*** | 1 |  |  |  |  |
| SZ | 0.171*** | -0.107*** | 0.369*** | -0.065*** | 0.285*** | -0.00500 | 0.041*** | 0.152*** | 0.406*** | -0.061*** | 1 |  |  |  |
| AGE | 0.230*** | 0.045*** | 0.342*** | -0.177*** | 0.345*** | -0.00900 | 0.070*** | 0 | 0.200*** | -0.034*** | 0.284*** | 1 |  |  |
| Market | -0.137*** | -0.125*** | -0.045*** | 0.064*** | -0.157*** | 0 | 0.046*** | -0.071*** | -0.145*** | 0.0150 | -0.187*** | -0.312*** | 1 |  |
| GDP | -0.176*** | -0.379*** | 0.107*** | 0.035*** | -0.094*** | 0.00800 | -0.049*** | -0.096*** | -0.160*** | 0.069*** | -0.126*** | -0.363*** | 0.387*** | 1 |
