# Supplementary material for: Unveiling the “Veil” of information disclosure: Sustainability reporting “greenwashing” and “shared value”
Source: PLoS One. 2023 Jan 18;18(1):e0279904. doi: 10.1371/journal.pone.0279904 (PMC9847897; doi:10.1371/journal.pone.0279904)
Supplement: S3 Table — (DOCX) [file pone.0279904.s003.docx]

**S3Table. VIF test results.**

| VARIABLE | VIF | 1/VIF |
| --- | --- | --- |
| SIZE | 2.20 | 0.454715 |
| ROA | 1.25 | 0.801420 |
| LEV | 1.72 | 0.579943 |
| GROWTH | 1.01 | 0.987072 |
| TAT | 1.06 | 0.945621 |
| TOP1 | 1.18 | 0.848184 |
| BZ | 1.68 | 0.595325 |
| IDR | 1.30 | 0.767761 |
| SZ | 1.37 | 0.731077 |
| AGE | 1.56 | 0.640983 |
| MARKET | 1.26 | 0.792194 |
| GDP | 1.62 | 0.616796 |
| Mean VIF | 1.43 |  |
