# Supplementary material for: Unveiling the “Veil” of information disclosure: Sustainability reporting “greenwashing” and “shared value”
Source: PLoS One. 2023 Jan 18;18(1):e0279904. doi: 10.1371/journal.pone.0279904 (PMC9847897; doi:10.1371/journal.pone.0279904)
Supplement: Attachment — Submitted filename: PONE-D-22-30440-Reviewer comments.docx [file pone.0279904.s004.docx]

**Unveiling the "Veil" of information disclosure: Sustainability reporting "Greenwashing" and "Shared value**

The underlined research topic “*Unveiling the "Veil" of information disclosure: Sustainability reporting "Greenwashing" and "Shared value*” is a novel part of the existing literature. The title of the article is remarkable, the presentation is good, and the article may provide several scientific justifications. Below are several suggestions which will increase the writer's input to technical research and probability.

Title: Title is good try to make it more attractive

Abstract: In general, the manuscript is straightforward. The theoretical framework is offered in the beginning and is quite helpful. It is clear that the authors have gone to great lengths to ensure that the reader is familiar enough with previous research to understand the rationale and methods of the present study. The methods can be used, however it would be helpful to have more background information on why they were selected. Those two quibbles aside, the conclusions are unanimous and compelling. The abstract would be more full with policy suggestions included.

The authors use a thorough approach, adding to the literature already present in this area. Overall, this is a well-written paper with intriguing implications for learning more about the origins, progressions, and longevity of eating disorders, but its relevance to nursing is unclear.

There was a failure to consider the study's consequences, and as a result, many concerns remained unresolved.

To provide a stronger theoretical contribution beyond that of previous research that employed an aggregate measure of job engagement, the author has to clarify and elaborate on why examining distinct dimensions of work engagement is useful.

Newer studies demonstrating the value of having a supportive superior should be added. Important normalcy assumptions should be clarified. In the abstract, they should briefly discuss the most significant results of their research. This is crucial for the audience to grasp the overall direction of the work. Also, the work has to detail its key contributions, such as new methods or insights.

Weakness in the literature review portion; author should offer theoretical and empirical context for important variables. The literature review primarily addresses the positive or negative impacts of investigated factors on environmental sustainability and energy usage and only gives abstract descriptions of prior studies. Adding solid theoretical explanations for underlying links between variables is recommended to strengthen this section. The following research could help strengthen this paper.

It is suggested that the article be revised since the section on policy suggestions is very simple and the author did not provide thorough policy recommendations based on the study results presented here. In order to strengthen the discussion sections, it is helpful to draw parallels between the current study and others that have been conducted on similar topics.

Although the study's findings and discussion are crucial, they take up too much space. If you can, cut out some of the tangential details that aren't essential to the study's argument.

Finally, it’s a nice attempt, the topic is of interest and has a wide range of policy implementation. I suggest "major revision” of the article for more practicability before "acceptance."
